# Sodium Metabisulfite-Induced Hematotoxicity, Oxidative Stress, and Organ Damage Ameliorated by Standardized *Ginkgo biloba* in Mice

**DOI:** 10.1155/2023/7058016

**Published:** 2023-10-10

**Authors:** Nancy Wambui Wairimu, Peninah Wairagu, Kennedy W. Chepukosi, George F. Obiero, Patrick W. Okanya, Alfred Orina Isaac, James Nyabuga Nyariki

**Affiliations:** ^1^Department of Biochemistry and Biotechnology, Technical University of Kenya, P. O. Box 52428, Nairobi 00200, Kenya; ^2^Department of Pharmaceutical Technology, School of Health Sciences and Technology, Technical University of Kenya, P. O. Box 52428, Nairobi 00200, Kenya

## Abstract

Sodium metabisulfite (SMB) is a biocide and antioxidant agent generally used as a preservative in food and beverage industries but can oxidize to harmful sulfite radicals. A standardized *Ginkgo biloba* (EGb-761) has demonstrated potent antioxidant and anti-inflammatory activities, which is beneficial for the treatment of diseases that exhibit oxidative stress and inflammation. The present study sought to investigate the putative ameliorative effects of EGb-761 against SMB-induced toxicity in mice. Thirty-two male Swiss white mice were randomized into control, SMB-treated, SMB + EGb-761-treated, and EGb-761-treated groups. EGb-761 (100 mg/kg/day) and SMB (98 mg/kg/day) were administered by gastric gavage for 40 days. Oral administration of EGb-761 restored SMB-induced decrease in body weight and prevented SMB-induced thrombocytopenia, leukocytosis, and anemia. Furthermore, EGb-761-treatment protected against SMB-induced liver and kidney injury depicted by decreased serum levels of aspartate aminotransferase (AST), alanine aminotransferase (ALT), alkaline phosphatase, bilirubin, creatinine, urea, uric acid, and albumin. Furthermore, EGb-761 treatment attenuated SMB-driven dyslipidemia and metabolic acidosis. Besides, EGb-761 supplementation abrogated SMB-driven oxidative stress as depicted by stabilized reduced glutathione (GSH) levels in the brain, liver, kidney, spleen, heart, and lungs. SMB induced a significant increase of tissue levels of malondialdehyde (MDA), serum nitric oxide (NO), interferon-gamma (IFN-*γ*) and tumor necrosis factor-*α* (TNF-*α*) which were abrogated by EGb-761 treatment. In conclusion, these results deepen our understanding of EGb-761 in light of various detrimental effects of SMB-driven toxicities. These findings provide a novel approach that can be optimized for preventing or treating exposure due to SMB toxicity.

## 1. Introduction

Sodium metabisulfite (SMB) is an inorganic chemical widely used as a preservative in the food, beverage, and pharmaceutical industries due to its ability to stop the growth of microorganisms and its antioxidant properties [1]. It is a sulfating agent that reacts with water to release toxic sulfur dioxide (SO_2_) when ingested or inhaled. Sulfites can be produced endogenously through the degradation of sulfur-containing amino acids such as cysteine and methionine or they can be obtained externally through food, drink, medicine, or from the environment by inhalation of polluted air [2]. The toxicity of sulfites is mitigated in vivo by sulfur oxidase that converts sulfites (SO_3_^2−^) to sulfates (SO_4_^2−^) [3]. The established and acceptable daily intake of ingested sulfites expressed as SO_2_ equivalents is 0.7 mg/kg body weight [4]. The toxic effects associated with SMB include male infertility [5], pneumonitis [6], increased lipid peroxidation [7], neurotoxicity [8], alterations in immunological, biochemical and hematological parameters [2], cytotoxicity of cells, and damage to the heart [1, 9].

Usage of SMB within the recommended concentrations is usually safe but it becomes toxic when used in excess or due to prolonged exposure. At higher concentrations, SMB acts as a prooxidant [7], and prolonged exposure may lead to deleterious effects on biological systems causing organ damage such as hepatotoxicity, nephrotoxicity, coronary artery disease, brain edema, and dementia [6]. The toxicity is caused by the generation of sulfites which are converted to sulfates by sulfur oxidase. Sulfates are oxidants that can be converted to reactive oxygen species and other SO_2_ free radicals responsible for various adverse effects [10]. From the literature review, it is evident that the concentration of SMB is not regulated in most countries and most flesh food vendors have the temptation to use copious amounts in order to extend the self-life of food.

*Gingko biloba* is a famous herbal medicinal plant that is cultivated because of its immense bioactive substances [11]. Ginkgo leaf extracts (EGb) have wide applications in alternative medicine, complementary medicine, food, and dietary supplements. The diverse EGb bioactive compounds include a variety of terpenoids, flavonoids, bioflavonoids, lignans, and organic acids [12–14]. A standardized *Ginkgo biloba* leaf extract (EGb 761) has been shown to contain multiple bioactive substances [15]. This standardized extract is by far among the most commonly used herbal medicines [12]. Its composition is majorly flavonoids and terpenoids including 22–27% ginkgo flavonoids mainly quercetin, kaempferol, and isorhamnetin, 5–7% terpene lactones of 3-4% ginkgolides A, B, and C; and 2.6–3.2% bilobalide and ginkgolic acid (<5 ppm) [16]. The EGb-761 is used for the treatment and management of neurological and cardiovascular diseases; its use has shown beneficial results in Alzheimer's dementia and ischemic stroke [17].

*Ginkgo biloba* leaf extracts possess powerful antioxidant properties that neutralize oxygen free radicals, the major cause of neurodegenerative diseases and aging [18]. In addition, EGb-761 can mitigate against lipid peroxidation by acting as a free radical scavenger and can reduce inflammation in diseases such as arthritis, irritable bowel syndrome, cancer, and heart diseases [19]. This is achieved via the reduction of inflammation by inhibiting the transcription of genes responsible for inflammatory responses and histamine release [18]. Besides, accumulating evidence suggests that *Ginkgo biloba* works by inhibiting inflammatory mediators such as NO, TNF-*α*, and inducible nitric oxide synthase (iNOS) [20]. Furthermore, ameliorative effects of *Ginkgo biloba* against lead and fluoride-induced toxicity have been documented [21]. These previous findings informed our use of *Ginkgo biloba* in this study, given that organ damage, inflammation, and oxidative stress are some of the features associated with SMB toxicity. Therefore, in the present study, the effects of EGb-761 treatment on various toxicities initiated by SMB exposure in mice were evaluated.

## 2. Materials and Methods

### 2.1. Experimental Design

The present study employed one control (naïve) group of mice and three treatment groups. In brief, thirty-two healthy male Swiss albino mice (5-6 weeks old) were randomly allotted into the four groups; each group containing 8 mice. Group 1 served as control and received distilled water and mice pellets. Group II mice received 98 mg/kg/day of sodium metabisulfite (SMB). Group III mice received 100 mg/kg/day of standardized *Ginkgo biloba* (EGb-76) and 98 mg/kg/day of SMB. EGb-76 is a well-characterized and standardized extract of *Ginkgo biloba* leaves that contain 24% flavone glycosides (primarily quercetin, kaempferol, and isorhamnetin) and 6% terpene lactones (2.8–3.4% ginkgolides A, B, and C and 2.6–3.2% bilobalide). Notably, ginkgolide B and bilobalide constitute approximately 0.8% and 3% of the total extract, respectively [22]. Group IV mice received 100 mg/kg/day of EGb-761 only. The mice were exposed to the treatments through oral administration using gastric gavage for 40 days. The animals were housed in sterile plastic cages under a controlled room temperature of 23–25°C and a 12 hour light/dark cycle and allowed to acclimatize for one week before the start of the experiments. The mice were fed on pellets (Unga feeds, Kenya) and had access to clean water ad libitum.

### 2.2. Preparation of Sodium Metabisulfite and *Ginkgo biloba*

Sodium metabisulfite 98 mg/kg/day (Sigma Aldrich, St Louis, MO) and standardized *Ginkgo biloba* extract (EGb 761) (eCRATER USA) were prepared fresh daily by dissolving them in sterile distilled water. The choice of 100 mg/kg/day dosage of EGb-761 was based on previous studies that showed potentiation of protective effects against lead-induced toxicity [23, 24].

### 2.3. Determination of Body Weight

The live body weights of animals from each experimental group were measured every three days throughout the experimental period. The body weight measurements were done using an analytical electronic balance (Mettler PM34, DoltaRange®).

### 2.4. Euthanization of Mice and Sample Collection and Preparation

After 40 days post-treatment, mice were sacrificed through euthanization with ketamine (50 mg/ml) and xylazine (100 mg/ml) (Merck KGaA, Darmstadt, Germany) in a ratio of 4 : 1 through intramuscular injection. Blood samples were collected intracardially from individual mice and placed in heparinized tubes for complete hemogram analysis and for biochemical analysis; blood was collected in sterile Eppendorf tubes. To obtain serum, blood in the Eppendorf tubes was left to settle for one hour at normal room temperature and centrifuged at 10,000 rpm at 4°C for 5 min (Centurion Scientific Ltd., K240R, UK). Mice were perfused with sterile PBS buffer after which the spleen, kidney, liver, lungs, heart, and brain were harvested and placed in Eppendorf tubes that were under dry ice. The snap-frozen whole brain, kidney, heart, lungs, spleen, and liver were homogenized on ice-cold water (4°C) in 0.5 ml of 0.25 M sucrose, 5 mM HEPES-Tris, pH 7.4, with protease inhibitor cocktail to a final concentration of 10% (w/v).

### 2.5. Hematological Determination and Biochemical Analysis

Analysis of individual blood samples from different experimental groups was done using an automated Beckman Coulter Counter (Benchman, Indianapolis, USA) to obtain full hemogram parameters. Serum levels of liver enzyme markers: alkaline phosphatase (ALP), alanine aminotransferase (ALT), aspartate aminotransferase (AST), gamma-glutamyl transferase (GGT), direct and total bilirubin, creatinine, urea, uric acid, albumin, total cholesterol, HDL, and triglycerides were assayed using an automatic analyzer (Integra 400 plus analyzer, Roche Diagnostics).

### 2.6. Cytokine ELISA

Serum levels of proinflammatory cytokines TNF-*α* and IFN-*γ* and IL-10 anti-inflammatory cytokine were measured by sandwich enzyme-linked immunosorbent assay (ELISA) (Thermo Fisher Scientific Inc., California, USA). The ELISA kits were used according to the manufacturer's detailed protocol. The ELISA optical reader (Multiskan EX-355, Thermo Electron Corporation, Waltham, Massachusetts, USA) was used to measure the absorbance that was set at 450 nm.

### 2.7. Reduced Glutathione (GSH) Assay

Reduced GSH content was determined by employing the method of Griffith [25] with some modifications. In brief, the brain, liver, kidney, heart, lungs, and spleen homogenates were mixed with a solution containing sulfosalicylic acid (4.31% (w/v)) and 0.25 mM EDTA. The GSH in the homogenates was determined chemically by reacting to GSH with Ellman's reagent (DTNB) and measuring the absorbance of the reaction product at 412 nm using a multidetection microplate reader (Biotek Synergy HT).

### 2.8. Nitric Oxide and Malondialdehyde Assay

Serum levels of nitric oxide (NO) were measured by the Griess assay kit (Sigma-Aldrich, St Louis, MO), which was used according to the manufacturer's instructions. NO production was quantified by measuring color change at 540 nm using a spectrometer (SpectraMax 340PC384, Molecular Devices, Sunnyvale, USA). Malondialdehyde (MDA) levels were measured by assays of thiobarbituric acid reactive species (TBARS) [26]. The quantification of the thiobarbituric acid reactive species (TBARS) was quantified by a spectrometer set at 535 nm.

### 2.9. Statistical Analysis

Statistical analysis was done using the GraphPad Prism software package (version 5.0). One-way ANOVA was done to compare the treatment groups with controls. For internal comparisons, Turkey's post-hoc test was used. The results were given as *a* ± SEM with significance set at *p* < 0.05.

## 3. Results

### 3.1. Effects of SMB and EGb-761 on Body Weight

**Figure 1:**
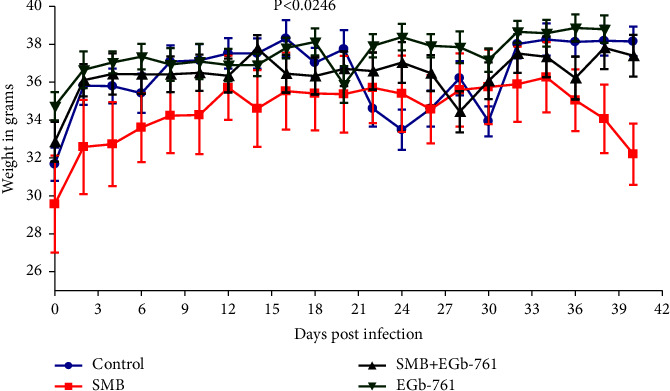
The effects of *Ginkgo biloba* on sodium metabisulfite-driven change in the general body weight. Change in body weight was analyzed using one-way ANOVA with Tukey's test for group comparisons.

There was a progressive increase in the live mean weight across all the groups of mice up to 18 days posttreatment. However, an intermediary decrease in body weight was observed in mice orally administered with SMB relative to other groups of mice (Figure 1). Notably, this decrease in general body weight was reversed by EGb-761 administration.

### 3.2. The Effects of EGb-761 on SMB-Induced Alterations of Red Blood Cells and Their Indices

**Figure 2:**
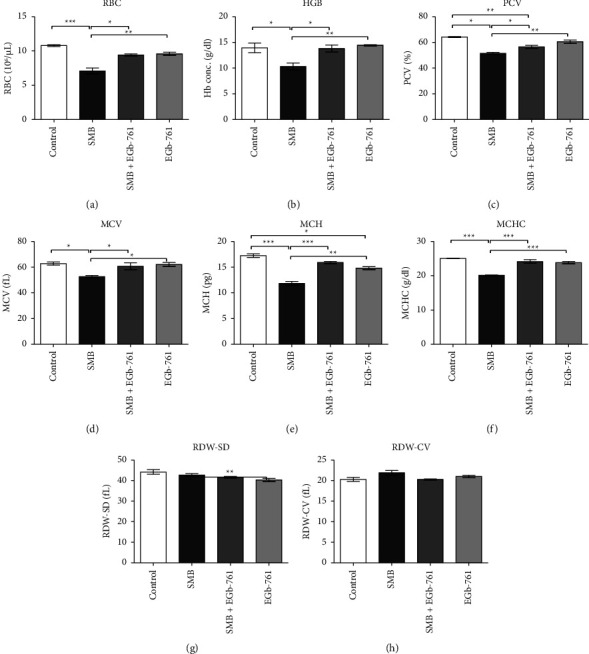
Effects of sodium metabisulfite and/or *Ginkgo biloba* administration on PCV, RBCs, and HGB and red blood cell indices in mice. Mean comparison procedures were done with one-way ANOVA with Tukey's multiple comparison post-hoc test. The results are expressed as ± SEM. The indicated level of significance was at ^*∗*^*p* < 0.05, ^*∗∗*^*p* < 0.01, and ^*∗∗∗*^*p* < 0.001.

Exposure of mice to SMB led to a significant reduction in the red blood cell (RBC), hemoglobin (HGB), and packed cell volume (PCV) (Figures 2(a)–2(c)), an indication of anemia. However, this suppression of RBC, HGB, and PCV levels was reversed by EGb-761 administration. Moreover, our findings reveal a significant decrease in the levels of mean corpuscular volume (MCV), mean corpuscular hemoglobin (MCH), and mean corpuscular hemoglobin concentration (MCHC) in the SMB-treated group of mice. The values of these parameters were reversed in the EGb-761-treated groups (Figures 2(d)–2(f)). On the contrary, the levels of red cell distribution width standard deviation (RDW-SD) and red cell distribution width coefficient of variation (RDW-CV) were comparable across all the treatment groups (Figures 2(g)- and 2(h)).

### 3.3. Effects of SMB and *Ginkgo biloba* on White Blood Cells and Their Subtypes

**Figure 3:**
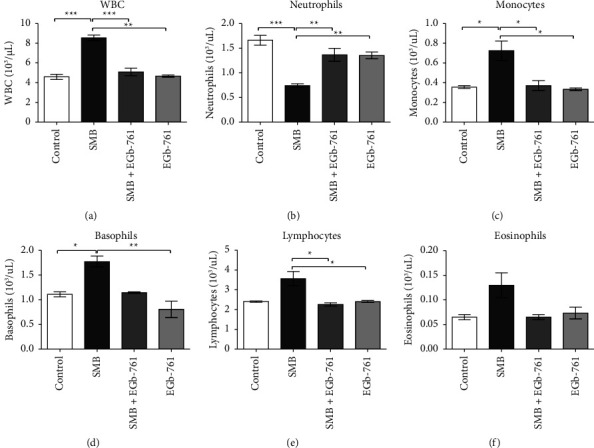
Effects of sodium metabisulfite and/or *Ginkgo biloba* administration on WBC and subtypes in mice. Mean comparison procedures were done with one-way ANOVA with Tukey's multiple comparison post-hoc test. The results are expressed as ± SEM. The indicated level of significance was at ^*∗*^*p* < 0.05, ^*∗∗*^*p* < 0.01, and ^*∗∗∗*^*p* < 0.001.

Exposure to SMB resulted in a significant increase in the levels of total white blood cell count (WBC) relative to those in the control group (Figure 3(a)). Remarkably, the administration of EGb-761 significantly restored SMB-driven leukocytosis. The results of WBC subtypes further confirmed that exposure to SMB resulted in a significant reduction in neutrophils (Figure 3(b)), which were upregulated in the EGb-761-treated mice. Furthermore, the levels of monocytes (Figure 3(c)), basophils (Figure 3(d)), and lymphocytes (Figure 3(e)) were significantly elevated following exposure to SMB and EGb-761-blocked SMB-driven elevation change. On the contrary, there was no statistically significant difference in the levels of eosinophils across all the treatment groups (Figure 3(f)).

### 3.4. Effects of Sodium Metabisulfite and *Ginkgo biloba* on Platelets and Their Indices

**Figure 4:**
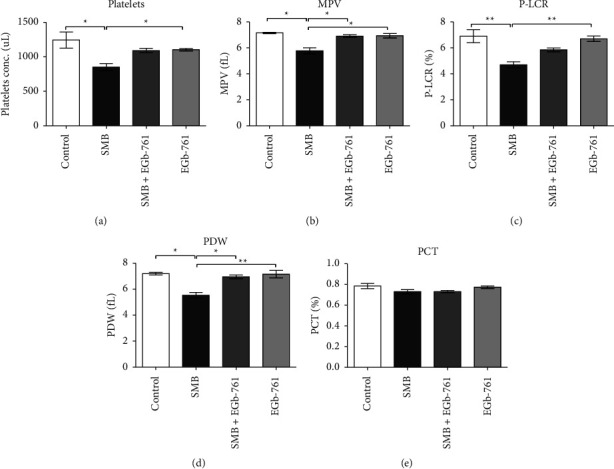
Effects of sodium metabisulfite and/or *Ginkgo biloba* administration on platelets and platelet subtypes in mice. Mean comparison procedures were done with one-way ANOVA with Tukey's multiple comparison post-hoc test. The results are expressed as ± SEM. The indicated level of significance was at ^*∗*^*p* < 0.05 and ^*∗∗*^*p* < 0.01.

Exposure of mice to SMB led to a significant decrease in platelet levels when compared to the control group (Figure 4(a)), denoting thrombocytopenia which was restored by EGb-761 administration. An analysis of the platelet indices showed SMB-driven downregulation of the mean platelet volume (MPV) (Figure 4(b)), platelet large cell ratio (P-LCR) (Figure 4(c)), and platelet distribution width (PDW) (Figure 4(d)), such changes were not present in the EGb-761-treated mice. The levels of plateletcrit (PCT) were unaffected by the treatments (Figure 4(e)).

### 3.5. Effects of Sodium Metabisulfite and *Ginkgo biloba* on Serum Lipid Levels

**Figure 5:**
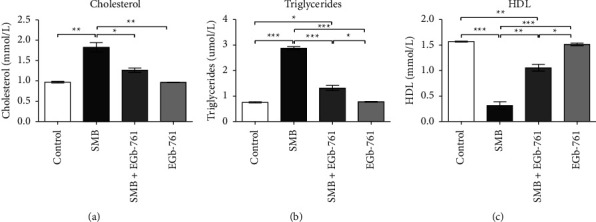
Effects of sodium metabisulfite and/or *Ginkgo biloba* administration on lipid profile. Mean comparison procedures were done with one-way ANOVA with Tukey's multiple comparison post-hoc test. The results are expressed as ± SEM. The indicated level of significance was at ^*∗*^*p* < 0.05, ^*∗∗*^*p* < 0.01, and ^*∗∗∗*^*p* < 0.001.

The SMB-administered mice showed a significant increase in the total cholesterol and triglyceride levels when compared to the control group (Figures 5(a) and 5(b), respectively). Notably, the administration of EGb-761 significantly attenuated the SMB-induced increase in total cholesterol and triglycerides. In contrast, the levels of high-density lipoprotein (HDL) were significantly decreased in SMB-treated mice when compared to control. Evidently, treatment with EGb-761 stabilized lipid levels across the board (Figure 5(c)).

### 3.6. Effects of SMB and *Ginkgo biloba* on Liver Function

**Figure 6:**
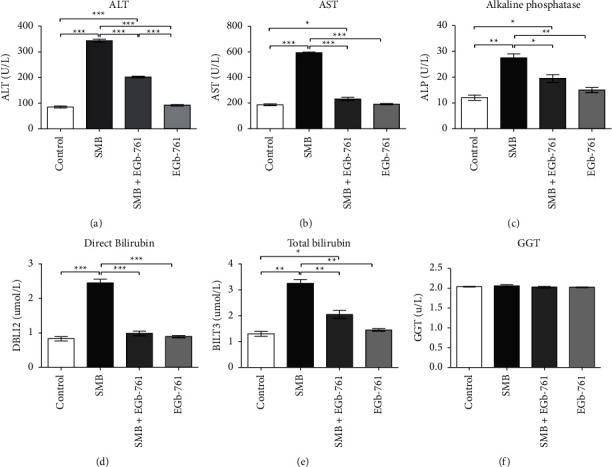
Effects of sodium metabisulfite and/or *Ginkgo biloba* on the levels of liver enzymes. Mean comparison procedures were done with one-way ANOVA with Tukey's multiple comparison post-hoc test. The results are expressed as ± SEM. The indicated level of significance was at ^*∗*^*p* < 0.05, ^*∗∗*^*p* < 0.01, and ^*∗∗∗*^*p* < 0.001.

Serum activities of ALT, AST, and ALP were significantly increased in the SMB-treated group compared to the control (Figures 6(a)–6(c)), indicative of active liver injury. Intriguingly, administration with EGb-761 protected mice against SMB-induced liver damage. In addition, our results indicated the serum levels of direct bilirubin and total bilirubin activities were significantly increased in mice exposed to SMB, which were reduced in the EGb-761-treated group (Figures 6(d)–and 6(e)). On the contrary, hepatic gamma-glutamyltransferase (GGT) was comparable in all the treated groups (Figure 6(f)).

### 3.7. The Impact of SMB and *Ginkgo biloba* Kidney Function

**Figure 7:**
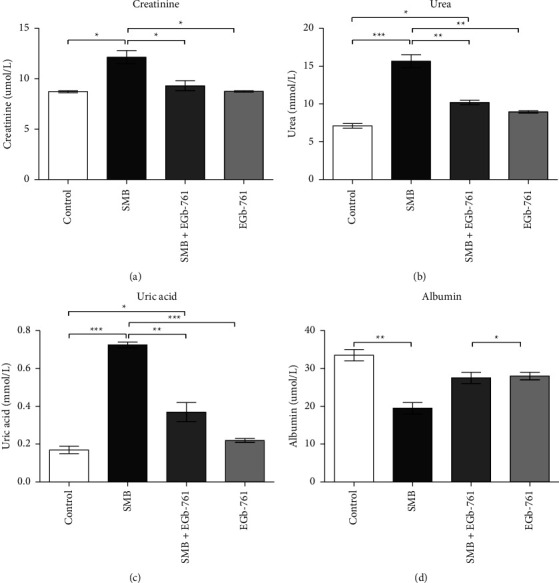
Comparison of the effect of sodium metabisulfite and/or *Ginkgo biloba* administration on the levels of creatinine, urea, uric acid, and albumin. Mean comparison procedures were done with one-way ANOVA with Tukey's multiple comparison post-hoc test. The results are expressed as ± SEM. The indicated level of significance was at ^*∗*^*p* < 0.05, ^*∗∗*^*p* < 0.01, and ^*∗∗∗*^*p* < 0.001.

Exposure of mice to SMB caused a significant increase in the serum levels of creatinine, urea, and uric acid in comparison to the controls (Figures 7(a)–7(c)). These heightened levels of creatinine, urea, and uric acid were reduced by treatment with EGb-761. Conversely, SMB caused a significant decrease in serum albumin levels; such changes were nullified by EGb-761 (Figure 7(d)).

### 3.8. The Impact of *Ginkgo biloba* on SMB-Driven Electrolyte Imbalance

**Figure 8:**
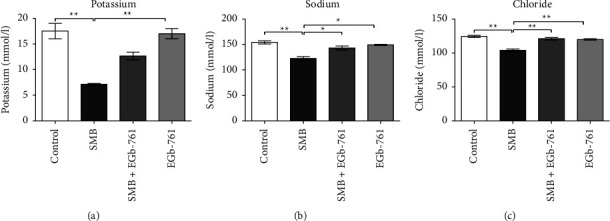
The effect of sodium metabisulfite and/or *Ginkgo biloba* administration on the levels of serum electrolytes. Mean comparison procedures were done with one-way ANOVA with Tukey's multiple comparison post-hoc test. The results are expressed as ± SEM. The indicated level of significance was at ^*∗*^*p* < 0.05 and ^*∗∗*^*p* < 0.01.

Exposure to SMB resulted in a significant decrease in the serum levels of potassium, sodium, and chloride ions (Figures 8(a)–8(c), respectively), indicative of SMB-driven active metabolic acidosis. In the presence of EGb-761, this phenomenon was alleviated.

### 3.9. The Impact of Sodium Metabisulfite and *Ginkgo biloba* on Malondialdehyde Levels

**Figure 9:**
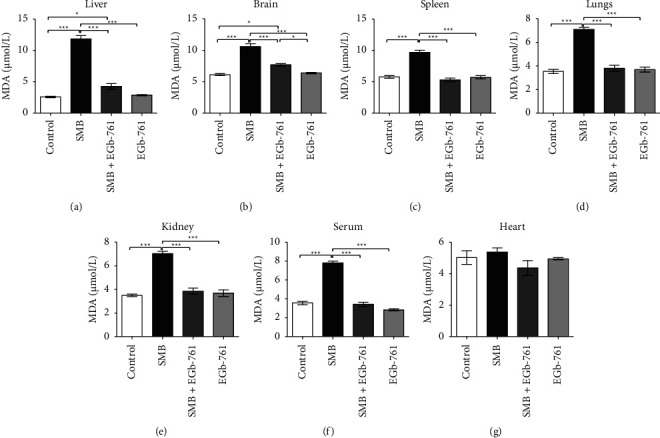
Comparison of the effect of sodium metabisulfite and/or *Ginkgo biloba* administration on the levels of malondialdehyde. Mean comparison procedures were done with one-way ANOVA with Tukey's multiple comparison post-hoc test. The results are expressed as ± SEM. The indicated level of significance was at ^*∗*^*p* < 0.05 and ^*∗∗∗*^*p* < 0.001.

Exposure to SMB resulted in a significant increase in the levels of malondialdehyde (MDA) in the liver, brain, spleen, lungs, kidney, and serum relative to the control (Figures 9(a)–9(f), respectively), depicting SMB-driven lipid peroxidation. Treatment with EGb-761 was able to alleviate this SMB-driven augmentation of MDA levels. In stark contrast, MDA levels in the heart were comparable for the normal control and mice that were administered with SMB and EGb-761 (Figure 9(g)).

### 3.10. Effects of Sodium Metabisulfite and *Ginkgo biloba* on the Levels of Reduced Glutathione

**Figure 10:**
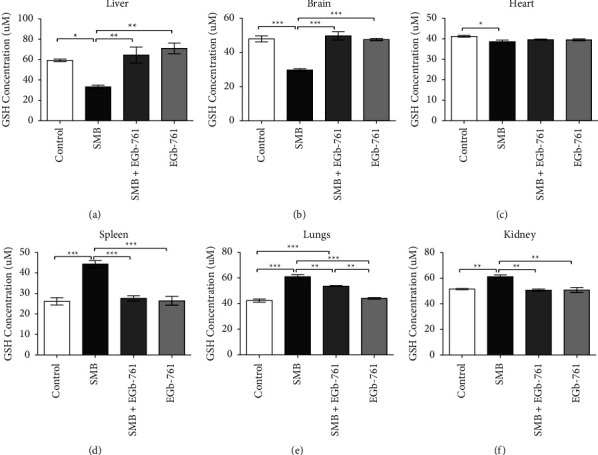
Comparison of the effect of sodium metabisulfite and/or *Ginkgo biloba* administration on the levels of cellular-reduced glutathione concentration in mice. Mean comparison procedures were done with one-way ANOVA with Tukey's multiple comparison post-hoc test. The results are expressed as ± SEM. The indicated level of significance was at ^*∗*^*p* < 0.05, ^*∗∗*^*p* < 0.01, and ^*∗∗∗*^*p* < 0.001.

The principal component analysis of reduced glutathione (GSH) revealed that exposure to SMB resulted in a significant depletion of liver, brain, and heart GSH levels when compared to the control group (Figures 10(a)–10(c)). Treatment of mice with EGb-761 significantly restored the levels of both hepatic and brain GSH levels. Furthermore, the results from the present study revealed that exposure to SMB caused a significant increase in the cellular GSH levels in the spleen, lungs, and kidney, which was restored in the presence of EGb 761 (Figures 10(d)–10(f)), reflecting the ameliorative effect of EGb-761 against SMB-induced oxidative stress in these organs.

### 3.11. Effects of Sodium Metabisulfite and *Ginkgo biloba* on Nitric Oxide Levels

**Figure 11:**
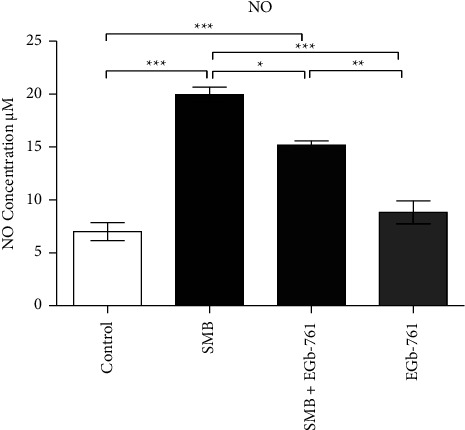
Effects of sodium metabisulfite and/or *Ginkgo biloba* administration on the levels of nitric oxide in mice. Mean comparison procedures were done with one-way ANOVA with Tukey's multiple comparison post-hoc test. The results are expressed as ± SEM. The indicated level of significance was at ^*∗*^*p* < 0.05, ^*∗∗*^*p* < 0.01, and ^*∗∗∗*^*p* < 0.001.

The levels of NO were significantly increased upon exposure of mice to SMB when compared to the control (Figure 11). Administration of EGb-761 decreased the SMB-induced increase of serum NO levels.

### 3.12. Effects of Sodium Metabisulfite and *Ginkgo biloba* on Cytokine Levels

**Figure 12:**
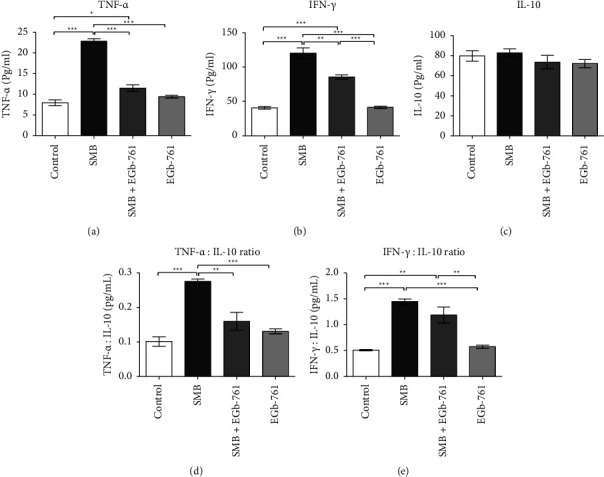
Comparison of the effect of sodium metabisulfite and/or *Ginkgo biloba* administration on the levels of the cytokines. Mean comparison procedures were done with one-way ANOVA with Tukey's multiple comparison post-hoc test. The results are expressed as ± SEM. The indicated level of significance was at ^*∗*^*p* < 0.05, ^*∗∗*^*p* < 0.01, and ^*∗∗∗*^*p* < 0.001.

Exposure to SMB caused a significant elevation of the proinflammatory cytokine tumor necrotic factor-alpha (TNF-*α*) and interferon-gamma (IFN-*γ*) (Figures 12(a) and 12(b)), indicative of inflammatory responses. Notably, this increase in proinflammatory cytokines was diminished in the presence of EGb-761. The serum levels of the anti-inflammatory cytokine interleukin-10 (IL-10) were comparable in all treated and control groups of mice (Figure 12(c)). An analysis of the ratios of the proinflammatory cytokines versus the anti-inflammatory cytokines TNF-*α*: IL-10 and IFN-*γ*-IL-10 revealed that the ratios were significantly higher in the SMB-exposed group of mice, which were reduced in the presence of EGb-761 (Figure 12(d) and 12(e)).

## 4. Discussion

Food preservatives are widely employed to circumvent food contamination due to microbial growth or undesirable chemical variations in packaged and stored food [27]. In the face of the well-known application of these preservatives in the beverage and food industry, the extent of their detrimental and toxic impact requires scrutiny. Sodium metabisulfite (SMB) is commonly used as a preservative in food processing and consumer products to combat the growth of microorganisms [1]. Excessive consumption of SMB, either through higher dosages or prolonged usage, causes many undesirable toxic and adverse effects [28].

It is increasingly evident that overuse of food additives as preservatives can significantly increase the development of human diseases [29]. *Ginkgo biloba* leaf extract is a potent antioxidant and anti-inflammatory agent. Besides this, it has been shown to have immunomodulatory effects as well as offer protection against various drug-induced organ pathologies. In this study, the role of standardized *Gingko biloba* leaf extract (EGb-761) in ameliorating sodium metabisulfite (SMB)-induced toxicities was evaluated. From our study, SMB induced hematotoxicity, oxidative stress, and disrupted immune function in mice. It was noted that most of these negative effects were markedly attenuated in the presence of EGb-761.

In this study, exposure to SMB-induced weight loss in mice relative to the control. Similar findings have been evident before in rats fed on high doses of SMB [2, 30], as well as pigs and rabbits [31, 32]. SMB-induced changes in feeding behavior contributed to the loss of weight. Furthermore, the weight loss following SMB exposure could be due to the toxicological effects of SMB [9]. Administration of EGb-761 significantly attenuated the SMB-induced weight loss. It is plausible to assume that the lipolysis-inducing property of EGb-761 may have contributed to this outcome as shown in a previous study [33].

Interference with the production of blood cells by chemical toxins is a common phenomenon with serious health implications. There was clear evidence of an SMB-driven decrease in the levels of RBCs, HGB, PCV, and the red cell indices, suggestive of anemia. These results corroborate a recently conducted study by Aslam [30], which showed that sulfites provoked a significant production of ROS that resulted in oxidative damage to the RBC membrane. In addition, RBC damage can be linked to lysis or feasible shrinkage of erythrocytes in blood [34]. The decline in the frequency of PCV may be associated with the reduction in the size of RBCs and the drop in the rate of synthesis of hemoglobin, which in turn controls the development and maturation of RBCs. Notably, MCH levels provide an indication of the actual content of hemoglobin in the RBC cytoplasm. Hence, MCV and MCHC levels are dependent upon the content of RBCs [35]. Possibly, the decrease in MCHC levels could be associated with the toxic effects of SMB in the bone marrow impairing its ability to produce hemoglobin at a requisite rate. Such effects on the hematopoiesis would affect the synthesis and production of all blood cells, consequently affecting the transport of oxygen and immune functions.

In the current study, it was demonstrated that EGb-761 reversed the SMB-induced anemia, indicative of a beneficial modulatory effect of EGb-761 on the hematopoietic system. These outcomes may be ascribed to the suppressive effect of SMB on the host hematopoiesis system. *Ginkgo biloba* has demonstrated a robust capacity to impede lipid peroxidation of RBC membranes, glutathione depletion, and methemoglobin development [36]. It is, therefore, plausible that these effects of EGb-761 may have played a fundamental role and perhaps protected the RBC from SMB-induced oxidant-driven damage.

White blood cells, also known as leukocytes, are highly versatile and play a critical role in coordinating and shaping the immune response. Any chemical-driven changes in WBCs would have a detrimental impact on immunity. In this study, exposure to SMB significantly increased the levels of WBCs, lymphocytes, basophils, and monocytes. Such findings had previously been shown by El-Kadi et al. [2]. From this study, SMB induced leukocytosis, perhaps due to stimulation of the lymphoproliferative responses by sulfites. We noted a remarkable SMB-driven depletion of neutrophils. Such suppression of neutrophils has the potential to predispose individuals to bacterial infections. Moreover, monocytosis and a significant increase in levels of basophils that was observed in mice exposed to SMB in the present study may predispose to inflammation-related ailments, given that effector basophils monocytes are implicated in inflammatory responses [37]. Remarkably, our findings demonstrated that treatment with EGb-761 can alleviate these detrimental effects due to its ability to stabilize WBC levels in the presence of SMB. The mechanism by which *Ginkgo biloba* ameliorates SMB-driven derangement of WBC and its subtypes could be multifactorial perhaps due to its antioxidant and anti-inflammatory activities [20].

Platelets in tandem with coagulation factors are indispensable during the thrombosis and hemostasis processes. Furthermore, platelets are involved in the inflammatory response and wound healing [38]. Therefore, a change in the content of platelet levels will critically interfere with these vital physiological and biochemical processes and present a higher threat for patients on blood thinning drugs [39]. To this end, exposure to SMB significantly suppressed platelet levels as well as MPV, P-LCR, and PDW, a clear indication of thrombocytopenia. From our study, treatment with EGb 761 significantly attenuated SMB-driven thrombocytopenia. These findings suggest that EGb-761 may have a modulatory role in the thrombocytosis and hemostasis processes. This phenomenon warrants further inquiry. Collectively, the results demonstrate that SMB negatively affected the hematopoietic processes. Importantly, EGb-761 supplementation reversed the SMB-induced hematotoxicity.

Lipid metabolism plays a critical role as an important source of macromolecular structures for the cell as well as a source of cellular energy. Consequently, alterations in lipids play a significant part in several pathophysiological disorders [40]. In the current study, exposure to SMB resulted in a significant elevation of total cholesterol and triglycerides with a concomitant decrease in the levels of high-density lipoproteins. This implies that people suffering from metabolic disorders and who are constantly exposed to SMB may aggravate the development of severe forms of the disease. In the presence of EGb-761, lipids metabolism was stabilized, demonstrating a possible modulatory role of *Ginkgo biloba* in lipid metabolism [33]. In a previous study, exposure to SMB was shown to influence lipid metabolism through increased release of free fatty acids (FFA) into the plasma [7], usually accompanied by inhibition of the enzyme lipase resulting in severe hypertriglyceridemia and hypercholesterolemia [7].

Sodium metabisulfite has been directly linked with severe liver damage in several studies [30]. In this study, liver enzymes that are important metric indexes of liver injury were measured to evaluate whether exposure to SMB affected liver function, and if administration of EGb-761 protected from SMB-driven liver damage. We report, that exposure to SMB significantly increased serum AST, ALT, and ALP, denoting liver damage. SMB-driven liver injury has been demonstrated in a prior study [30]. The levels of these enzymes are elevated and released into the plasma under hepatocellular membrane stress, depicting liver injury [2]. It is noteworthy that in the presence of EGb-761, the SMB-driven elevation of liver enzymes was abrogated.

Bilirubin is a byproduct released following the prompt destruction of the RBC. Heightened levels of bilirubin and its buildup in the hepatocellular environment result in inflammation and organ injury [41]. SMB induced significant elevation of bilirubin. Notably, SMB-driven elevation of bilirubin was attenuated by oral administration of EGb-761. Since bilirubin is a product of RBC breakdown, the results point to a possible novel protective effect on the liver and RBCs.

Additional investigations determined the integrity of renal function in the presence of SMB and EGb-761. Creatinine and urea are important markers of kidney function, and their upsurge or reduction mirrors a dysfunction of the kidney [42]. Indeed, the breakdown of liver protein compounds has been implicated in the intensification of urea and creatinine levels in animal models [2]. These increased levels of urea and creatinine may be linked to the kidney injury that was observed in this study as revealed by a significant increase of serum creatinine and urea levels in mice exposed to SMB. The observed upsurge in serum levels of uric acid may be attributed to the reduction in urinary excretion of the metabolites. Herein, we report that SMB-driven kidney injury was attenuated by administration of EGb-761, inferring protection against nephrotoxicity. This result is in harmony with the previous work where *Ginkgo biloba* was observed to have a renoprotective effect against cisplatin-induced nephrotoxicity and renal damage due to ischemic reperfusion [43].

Uric acid is the end product of purine degradation [44]. Hyperuricemia has been implicated as a driving force behind cognitive impairment, cardiovascular maladies, and oxidative stress [45]. In the present study, mice exposed to SMB had significantly high uric acid levels, which is in agreement with prior studies [30]. Augmented levels of uric acid are associated with increased production of proinflammatory cytokines such as TNF-*α*, IL-1*β*, and IL-6 [46]. Thus, SMB-driven elevation of uric acids may in part contribute to inflammation of the kidney and liver, which will directly exacerbate the pathophysiology of these organs. We report that administration of EGb-761 significantly reduced these elevated levels of uric acid. This modulation of uric acid by EGb-761 may attenuate SMB-driven toxicity and inflammation of the kidney and liver.

Serum albumin is a crucial protein with a vital physiological role and antioxidant activities [47]. It is produced in the liver and can be used as a biomarker for early liver impairment and chronic liver diseases [48]. In the current study, we assessed serum albumin levels in mice exposed to SMB and *Ginkgo biloba*. Exposure of mice to SMB resulted in a significant decrease in the serum levels of serum albumin, indicative of liver impairment. It was clear that EGb-761 treatment of SMB-exposed mice significantly increased the levels of serum albumin, demonstrating protection against liver injury.

Metabolic acidosis is a common condition characterized by a fall in pH by several toxins [49]. In addition, metabolic acidosis is usually an indication of serious pathological states. Thus, understanding how exposure to toxicants such as SMB disrupts the physiological pH buffering system is important. A significant sodium metabisulfite-driven metabolic acidosis was noted in the present study as demonstrated by a significant decrease in serum levels of potassium, sodium, and chloride ions. Metabolic acidosis often arises, partly when there is the acceleration of movement of sodium ions into the cell in response to severe intracellular acidosis with the potential for cell dysfunction. Notably, SMB-driven metabolic acidosis was prevented by EGb-761 administration. This observation may be ascribed to its role in the maintenance of the membrane ultrastructure against lethal effects associated with the generation of free radicals as well as protection against modulation of enzymatic systems and ionic pumps [50]. Nevertheless, the mechanisms by which EGb-761 regulates metabolic acidosis merit further investigations.

Oxidative stress is a phenomenon that is known to underlie or even aggravate the pathogenesis of several disease processes including but not limited to cancer, atherosclerosis, neurodegenerative diseases hypertension, diabetes mellitus, cardiovascular disease, atherosclerosis, reproductive system diseases, and aging [51]. Moreover, elevated levels of lipid peroxides resulting from augmented production of free radicals may be important molecular mechanisms for sodium metabisulfite-associated deleterious effects [52]. The uncontrolled oxidation of sulfite into sulfite free radicals may trigger sulfite‐driven lipid peroxidation [53]. In addition, uncontrolled lipid peroxidation may drive the production of malondialdehyde (MDA). MDA is a critical marker of lipid peroxidation [54]. In the current study, exposure of mice to SMB led to an increase in tissue and serum MDA, indicating the presence of lipid peroxidation. Remarkably, the administration of EGb-761 attenuated an SMB-driven increase in MDA levels, a protective effect that can be attributed to its antioxidant properties. These results are in line with published data [55], which demonstrated the ability of EGb-761 to scavenge free radicals with concomitant reduction in MDA associated with lipid peroxidation.

Indeed, oxidative stress is known to cause damage to important cellular biomolecules [56]. Besides, accumulating evidence has shown that due to its sulfites and its derivatives, SMB can cause oxidative stress as a result of sulfite oxidation and DNA damage in vital organs such as the liver, brain, lung, and spleen [57].

In the physiological environment, cells cope with excessive ROS using highly versatile and potent endogenous antioxidant enzymes consisting of GSH, superoxide dismutase (SOD), glutathione peroxidase, and catalases. Depletion of these important antioxidant systems elicits elevation of lethal ROS, thus causing oxidative stress. Consequently, levels of antioxidant enzymes such as GSH are very good indicators of oxidative stress. In agreement with the earlier findings [58], SMB administration significantly depleted GSH levels in the liver, brain, and heart with elevation of GSH being observed in the spleen, lungs, and kidney, indicative of oxidative stress. Depletion of GSH is a clear indication of overwhelming and lethal oxidative stress levels, whereas it is characteristically associated with an initial response to rising levels of oxidative stress [59–61].

*Ginkgo biloba* has been proposed as an antioxidant agent in numerous studies [62–64]. Recently, it has been shown to exert its effect directly by scavenging ROS or elevating the expression of genes encoding antioxidant enzymes [65]. Moreover, both in vitro and in vivo studies have shown that the antioxidant property of *Ginkgo biloba* is associated with its flavonoid components, such as kaempferol and quercetin that suppress ROS [66]. We also assessed the antioxidant activity of EGb-761 using GSH levels following exposure of mice to SMB. Remarkably, this is the first study demonstrating that EGb-761 administration resulted in the assuaging of oxidative stress by SMB in vital organs such as brain, liver, kidney, lungs, spleen, and heart.

The induction of nitric oxide synthase (iNOS) leads to the elevation of nitric oxide (NO), leading to the inhibition of the respiratory chain and a reduction in ATP formation [67]. Besides, the excessive production of NO is the hallmark of different pathological disorders [68]. Specifically, NO facilitates the generation of lethal reactive metabolite, peroxynitrite (ONOO^−^) [69], which nitrates vital lipids, nucleic acids, proteins, and/or enzymes in the physiological environment of vital organs, altering their structure and rendering them dysfunctional. Moreover, NO-mediated inflammatory processes and oxidative stress events have also been outlined [70]. Given that many toxic chemicals induce inflammation and oxidative stress, the identification of novel compounds that are good candidates for the downregulation of inflammatory mediators is of great significance. Herein, we found out that exposure of mice to SMB resulted in a significant increase in the serum levels of NO. Remarkably, EGb-761 nullified SMB-induced elevation of NO. Overwhelming evidence has demonstrated that *Ginkgo biloba* protects cells from NO-induced neurotoxicity and several inflammatory mediators [71]. Thus, the protective ability of EGb-761 against SMB, noted in the current study, may be associated with its proven anti-inflammatory and antioxidant activities [53].

The body gets rid of detrimental stimuli such as toxic compounds and invading pathogens by mounting strong immune responses [72]. Exposure to SMB has been demonstrated to enhance the pyroptosis process which ultimately results in increased amounts of proinflammatory cytokines IL-1*β* and IL-18 [73]. Impairment of these processes due to continuous exposure to sodium metabisulfite may cause chronic inflammation. Our study revealed that exposure to SMB resulted in a significant increase of serum TNF-*α* and IFN-*γ*, indicative of active SMB-induced inflammation. In the presence of EGb-761, SMB-induced elevation of these proinflammatory cytokines was abrogated. It is well documented that a balance between anti-inflammatory and proinflammatory cytokines defines the inflammatory state of the cellular environment. Thus, determining the ratio between the proinflammatory and anti-inflammatory cytokines may help to determine the degree of inflammatory status due to SMB exposure. Furthermore, we demonstrate a noticeable imbalance of proinflammatory and anti-inflammatory cytokines in an SMB-administered group of mice that reflects aggravated inflammation. In addition, the anti-inflammatory effects of EGb-761 treatment were also confirmed by a stable balance between proinflammatory and anti-inflammatory cytokines, once again showing the anti-inflammatory action of EGb-761. The anti-inflammatory properties of *Ginkgo biloba* have been proven in several studies [20, 22, 74]. Accordingly, it has been shown that administration of *Ginkgo biloba* plays an important role in the resolution of inflammation through the reduction of tumor necrosis factor (TNF-*α*) and interleukin 1*β* (IL-1*β*), while enhancing the level of anti-inflammatory cytokine interleukin 10 (IL-10) [75]. Its anti-inflammatory properties are attributed to various flavone glycosides and terpenoids contained in it.

## 5. Conclusion

In summary, the present study demonstrates for the first time that oral administration of standardized *Ginkgo biloba* (EGb-761) attenuated SMB-induced alteration of hematological parameters, metabolic acidosis, inflammatory responses, oxidative stress, and organ [76] damage. Arguably, because exposure to SMB results in varied detrimental effects, our findings have significant and immediate clinical implications.

## Data Availability

The data used to support the findings of this study are available on request from the authors.
